# Molecular determinants of vascular transport of dexamethasone in COVID-19 therapy

**DOI:** 10.1107/S2052252520012944

**Published:** 2020-09-29

**Authors:** Ivan G. Shabalin, Mateusz P. Czub, Karolina A. Majorek, Dariusz Brzezinski, Marek Grabowski, David R. Cooper, Mateusz Panasiuk, Maksymilian Chruszcz, Wladek Minor

**Affiliations:** a Department of Molecular Physiology and Biological Physics, University of Virginia, 1340 Jefferson Park Avenue, Charlottesville, VA 22908, USA; b Center for Biocrystallographic Research, Institute of Bioorganic Chemistry, Polish Academy of Sciences, 61-704 Poznan, Poland; c Institute of Computing Science, Poznan University of Technology, 60-965 Poznan, Poland; d Department of Clinical Medicine, Medical University of Bialystok, 15-089 Bialystok, Poland; e Department of Chemistry and Biochemistry, University of South Carolina, Columbia, SC 29208, USA

**Keywords:** COVID-19, dexamethasone, SARS-CoV-2, coronavirus, drug transport, albumin

## Abstract

Dexamethasone, a widely used corticosteroid, has recently been reported as the first drug to increase the survival chances of patients with severe COVID-19. Therapeutic agents, including dexamethasone, are mostly transported through the body by binding to serum albumin. Here, the first structure of serum albumin in complex with dexamethasone is reported. Dexamethasone binds to drug site 7, which is also the binding site for commonly used nonsteroidal anti-inflammatory drugs and testosterone, suggesting potentially problematic binding competition. This study bridges structural findings with an analysis of publicly available clinical data from Wuhan and suggests that an adjustment of the dexamethasone regimen should be further investigated as a strategy for patients affected by two major COVID-19 risk factors: low albumin levels and diabetes.

## Abstract 2

The structure of serum albumin in complex with dexamethasone reveals why the drug may not always help COVID-19 patients.

## 1. Introduction   

On 16 June 2020, collaborators in the RECOVERY (Random­ized Evaluation of COVID-19 Therapy) trial reported that a daily dose of 6 mg dexamethasone reduced deaths among COVID-19 patients receiving respiratory interventions (Ledford, 2020 ▸; RECOVERY/University of Oxford, 2020 ▸). This widely available steroid has been shown to cut deaths by approximately 30% for patients who were on ventilators and by 20% for those who were receiving oxygen therapy but were not on ventilators. Decreased mortality with dexamethasone treatment was later confirmed by two other studies, which employed significantly higher doses of dexamethasone (Sterne *et al.*, 2020 ▸). Dexamethasone is a corticosteroid that exhibits potent anti-inflammatory and immunosuppressant effects. Since the 1960s, it has been used to treat many conditions, including severe pneumonia, rheumatic problems, skin diseases, severe allergies, asthma, chronic obstructive lung disease, brain swelling and many others (Yuanjing *et al.*, 2009 ▸; https://www.drugbank.ca/drugs/DB01234). Dexamethasone suppresses the immune system by binding to the glucocorticoid receptor, which up-regulates the expression of anti-inflammatory proteins and down-regulates the expression of pro-inflammatory proteins (Rhen & Cidlowski, 2005 ▸). The reduced inflammation provides some relief for patients whose lungs have been devastated by the overactive immune response that accompanies severe cases of COVID-19, but shows no benefit for patients who do not require respiratory support (RECOVERY Collaborative Group *et al.*, 2020 ▸). Along with anti-inflammatory action, steroids can also reduce airway spasm and obstruction in patients who have asthma or chronic obstructive pulmonary disease: groups of patients that are at especially high risk (Alangari, 2014 ▸). In the RECOVERY trial, the therapeutic effect of dexamethasone appeared to depend on ‘using the right dose, at the right time, in the right patient’ (RECOVERY Collaborative Group *et al.*, 2020 ▸). A meta-analysis of seven randomized clinical trials that evaluated the efficacy of corticosteroids did not find evidence that a higher dose of corticosteroids was associated with a greater benefit than a lower dose of corticosteroids (Sterne *et al.*, 2020 ▸). In this study, we propose that there may be another important factor which might influence the effectiveness of dexamethasone and should be investigated: its vascular transport.

Dexamethasone is primarily delivered throughout the body by serum albumin; about 77% of dexamethasone in the blood is bound to plasma proteins, mostly to serum albumin (https://www.drugbank.ca/drugs/DB01234; Zhao *et al.*, 2009 ▸; Cummings *et al.*, 1990 ▸; Naik *et al.*, 2010 ▸). Serum albumin has a typical blood concentration of 35–55 g l^−1^ (600 µ*M*) and constitutes up to 55% of the total plasma proteins (Rifai, 2018 ▸). During its month-long lifetime, an albumin molecule makes nearly 15 000 trips around the body, facilitating the vascular transport of hormones, metals, fatty acids and drugs (Peters, 1995 ▸). The extensive drug-binding capacity of albumin is enabled by its high concentration and by the presence of at least ten distinct drug-binding sites on the molecule (Czub *et al.*, 2020 ▸). Preferred binding sites on albumin are known for only 32 drugs, including ibuprofen, warfarin, cetirizine, naproxen, diazepam, etodolac and halothane (Czub *et al.*, 2020 ▸). The free fraction of dexamethasone in blood and its distribution over time can be affected by several factors, including a decreased albumin plasma level (Roberts *et al.*, 2013 ▸), drugs competing for the same binding sites (Trainor, 2007 ▸; Bohnert & Gan, 2013 ▸; Sułkowska *et al.*, 2004 ▸; McElnay & D’Arcy, 1983 ▸) and albumin glycation (Anguizola *et al.*, 2013 ▸). Here, we propose that the inconsistent therapeutic action of dexamethasone in COVID-19 may be partially explained by differences in its vascular transport. We report the molecular structure of albumin in complex with dexamethasone, provide new insights into the mechanism of its transport, and analyze serum albumin and glucose levels in publicly available clinical data (Yan *et al.*, 2020 ▸). We hypothesize that compromised vascular transport of dexamethasone may limit its effectiveness in COVID-19 therapy, and propose that adjusted dexamethasone regimens should be further investigated.

## 2. Materials and methods   

### 2.1. Materials   

Equine serum albumin (ESA) was purchased from Equitech-Bio, Kerrville, Texas, USA (catalog No. ESA62; ≥96% purity) as a lyophilized powder and was purified further as described in Section 2.2. Dexamethasone was purchased from Sigma–Aldrich, St Louis, Missouri, USA (catalog No. D1756; ≥98% purity). The purity of all reagents was as reported by the vendors.

### 2.2. Protein purification and crystallization   

ESA was dissolved in a buffer consisting of 10 m*M* Tris pH 7.5, 150 m*M* NaCl. Size-exclusion chromatography using a Superdex 200 column attached to an ÄKTA FPLC (GE Healthcare) was used for further purification and to separate the dimeric and monomeric fractions of ESA. The purification buffer was the same as the buffer in which the protein was dissolved. The absorbance at 280 nm, as measured with a NanoDrop 2000 (Thermo Scientific), was used to estimate the protein concentration using the extinction coefficient (∊_280-ESA_ = 27 400 *M* ^−1^ cm^−1^) and molecular weight (MW_ESA_ = 65 700 Da). The collected fractions of monomeric ESA were concentrated to 15 mg ml^−1^ using an Amicon Ultra Centrifugal Filter (Millipore Sigma, catalog No. UFC903024) with a molecular-weight cutoff (MWCO) of 30 kDa. Protein crystallization was performed in 15-well hanging-drop plates (EasyXtal 15-Well Tools, Qiagen). Prior to crystallization, dexamethasone powder at a tenfold molar excess was added to the concentrated protein solution (15 mg ml^−1^ ESA) in purification buffer. The mixture was incubated for 60 min at room temperature and was then used for crystallization with some of the undissolved powder in suspension. Aliquots of 1 µl of the mixture were combined with 1 µl reservoir solution (1.8 *M* ammonium dihydrogen citrate pH 7.0). The harvested crystals were flash-cooled in liquid nitrogen using a 1:1 mixture of Paratone-N and mineral oil as a cryoprotectant.

### 2.3. Data collection and structure determination   

Diffraction data were collected at 100 K on the 21-ID-F beamline at the Advanced Photon Source, Argonne National Laboratory using a MarMosaic 225 mm CCD detector. The collected data were processed, integrated and scaled with *HKL*-3000 using corrections for radiation decay and anisotropic diffraction (Otwinowski & Minor, 1997 ▸; Minor *et al.*, 2006 ▸; Borek *et al.*, 2010 ▸). The resolution cutoff and the number of images to be included in the final data set were chosen based on the values of CC_1/2_, 〈*I*〉/〈σ(*I*)〉, completeness and *R*_meas_. The initial phases were determined by molecular replacement using PDB entry 3v08 (Majorek *et al.*, 2012 ▸) as the template. The structure was refined with H atoms in riding positions using *HKL*-3000, which interacts with *REFMAC* and other programs from the *CCP*4 package (Vagin & Teplyakov, 2010 ▸; Murshudov *et al.*, 2011 ▸; Winn *et al.*, 2011 ▸). *Coot* (Emsley & Cowtan, 2004 ▸; Emsley *et al.*, 2010 ▸) was used for manual correction of the model. The protein model was placed in the standardized position in the unit cell using the *ACHESYM* server (Kowiel *et al.*, 2014 ▸). TLS groups were determined and set up with a standalone version of the *TLS Motion Determination* server (Painter & Merritt, 2006 ▸). The use of TLS parameters was justified by a significantly improved *R*_free_ and the Hamilton *R*-factor ratio test (Merritt, 2012 ▸) as implemented in *HKL*-3000. The structure refinement and model completion followed recently published guidelines (Shabalin *et al.*, 2018 ▸; Majorek *et al.*, 2020 ▸), thus avoiding the problems that have been observed for some SARS-CoV-2 drug-target models (Wlodawer *et al.*, 2020 ▸). Both *MolProbity* (Chen *et al.*, 2010 ▸) and the wwPDB validation server (Gore *et al.*, 2017 ▸) were used for model validation. *PyMOL* (version 1.5.0.3; Schrödinger) was used for the preparation of structural figures. All experimental steps were tracked using LabDB (Zimmerman *et al.*, 2014 ▸). *Molstack* (Porebski *et al.*, 2018 ▸, 2020 ▸) was used for interactive visualization of the model and the electron density maps online. Diffraction images were deposited in the Integrated Resource for Reproducibility in Macromolecular Crystallography at http://proteindiffraction.org (Grabowski *et al.*, 2016 ▸, 2019 ▸) and are available at https://doi.org/10.18430/m3.irrmc.5571. The atomic coordinates and structure factors for the structure were deposited in the Protein Data Bank with accession code 6xk0. Statistics for diffraction data collection, structure refinement and structure quality are presented in Table 1 ▸.

The raw diffraction data set for the albumin–dexamethasone structure presented here was collected nine years ago as part of the New York Structural Genomics Research Consortium project. The refinement of this particular structure had to wait owing to an avalanche of other projects, only to become more consequential now that the RECOVERY group has announced that dexamethasone can be used to treat COVID-19 patients. This example shows that sometimes the importance of basic science is not immediately apparent, but the questions that it can answer can one day become of tremendous value. The use of LabDB (Zimmerman *et al.*, 2014 ▸) was essential for locating the appropriate data set and all of the information about sample preparation and the X-ray experiment, demonstrating that a reliable data management system with extensive descriptions of experiments has a vital advantage for research laboratories.

### 2.4. Clinical data of COVID-19 patients   

The albumin and glucose levels of patients from Tongji Hospital, Wuhan, People’s Republic of China were taken from the data set published by Yan *et al.* (2020 ▸) available at https://github.com/HAIRLAB/Pre_Surv_COVID_19. The data set was made public under the MIT License. As described in Yan *et al.* (2020 ▸), the blood-test results of the patients were collected between January 10 and February 18, 2020. The original data set describes 375 patients, but here we analyze only the 373 patients for whom albumin levels were reported. Most of these patients (356 out of 373) had multiple blood samples taken throughout their stay in the hospital. If not stated otherwise in the text, we used the last sample taken to calculate statistics, as it most accurately matches the patient’s outcome (died or survived). However, the median changes in the albumin levels of patients over time were very small [Fig. 3(*c*)], and statistics obtained using other samples would be almost identical. Reproducible analysis scripts are available at https://github.com/dabrze/covid_albumin_levels.

### 2.5. Statistical methods   

The differences in sample means were assessed using two-tailed Welch’s *t*-tests at significance level α = 0.05. Prior to assessing the significance of the differences, the samples were checked for normality using the Kolmogorov–Smirnov test with α = 0.05 and visually inspected using Q–Q plots. To calculate the correlation between the admission and final albumin levels, we used the Pearson product–moment correlation coefficient, two-tail-tested against a *t* distribution with *n* − 2 degrees of freedom (df_Died_ = 346, df_Survived_ = 396) at α = 0.05; patients with only one albumin level available were excluded from the calculation.

Logistic regression models were used to estimate the association between patients’ albumin levels and their survival or death. We chose logistic regression as it is a multivariable method that is routinely used in statistical analyses in the medical literature (Bagley *et al.*, 2001 ▸). It analyzes the relation between several predictor variables and one outcome variable. The result of the analysis is a set of coefficients that describe the relative contribution of each predictor to the outcome variable, while controlling for the influence of other predictors. These coefficients are typically transformed to represent the odds (ratio of probabilities) of the outcome. Therefore, logistic regression can help to answer questions about how strongly different variables are associated with a given outcome.

In the regression models described in this paper, the analyzed predictors were the albumin level, glucose level, age and gender of the patient, whereas the outcome variable was the status of the patient (died or survived). The albumin regression model was adjusted to take into account the remaining predictors (age, gender and glucose level) as confounding factors. This means that the interactions between the predictors were checked to assess whether one predictor is correlated with another and whether this changes the significance of the predictor or its effect on the outcome. All confounders were checked for potential effect modification, but no effect modification was found, as all interaction terms exhibited *p* > 0.2. Detailed odds ratios with confidence intervals and *p*-values are presented in Supplementary Table S1. It should be noted that the analyzed data originate from blood samples of patients admitted to the hospital, not from a controlled trial. Therefore, the results should be interpreted in terms of correlation (association), not causation.

## 3. Results and discussion   

### 3.1. Structure of serum albumin in complex with dexamethasone   

The crystal structure of equine serum albumin (ESA) in complex with dexamethasone was determined at 2.4 Å resolution. During model building, the polypeptide chain was almost entirely completed, except for the first three N-terminal residues, which were not located in the electron density maps. Albumin consists of three homologous domains: I (residues 1–195), II (residues 196–383) and III (residues 384–585). Each domain contains two subdomains (A and B) composed of six and four α-helices, respectively. The overall fold of ESA in this structure is essentially identical to that in previously published structures of ESA and human serum albumin (HSA; Supplementary Table S2). According to the *DALI* server (Holm, 2019 ▸), the closest structure is the ligand-free structure of ESA (PDB entry 3v08; Majorek *et al.*, 2012 ▸), with an r.m.s.d. of 0.4 Å.

**Figure 1:**
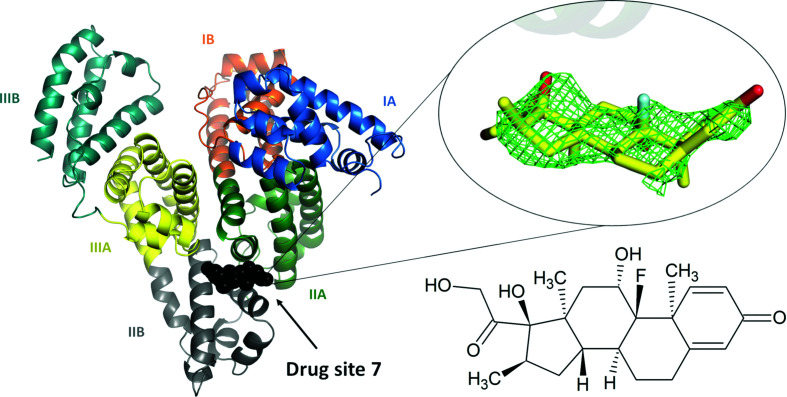
The overall structure of the ESA–dexamethasone complex. The electron density observed for dexamethasone in DS7 is shown as a green mesh (*mF*_o_ − *DF*_c_ map, calculated after ten refinement cycles without the ligand, r.m.s.d. 2.5). Albumin subdomains are shown in different colors and labeled with Roman numerals and letters (*e.g.* IA). The dexamethasone molecule is shown in stick representation with C atoms in yellow, O atoms in red and the F atom in cyan. The chemical structure of dexamethasone is displayed in the same orientation as the stick representation. The electron density, including the OMIT maps, and the model can be inspected interactively at https://molstack.bioreproducibility.org/project/view/gmvG1L8c66YgPpgqS0Zm/.

The electron density maps are consistent with one dexamethasone molecule bound in drug site 7 (DS7), which is located in domain II between subdomains IIA and IIB (Fig. 1 ▸). The drug-site numbering is according to the recently expanded nomenclature (Czub *et al.*, 2020 ▸), which was built upon the previously used site names (Zsila, 2013 ▸; Handing, Shabalin, Szlachta *et al.*, 2016 ▸). Notably, DS7 was always unoccupied in the various ESA structures determined in our laboratory that did not have an intentionally added drug (Majorek *et al.*, 2012 ▸; Handing *et al.*, 2016 ▸). During refinement, a different orientation of dexamethasone was tried as an alternative, in which the drug was rotated by 180° along the axis perpendicular to its rings. However, in the alternative orientation one of the four rings of dexamethasone was outside strong electron density, and the compound did not form any hydrogen bonds to the protein, clearly supporting the chosen conformation. The mode of binding of dexamethasone in the chosen orientation is very similar to that observed in the very well defined electron density for the structurally similar testosterone (Czub *et al.*, 2019 ▸). ADP, RSR and RSCC values for the dexamethasone molecule are reported in Table 1 ▸. The ADP values of the dexamethasone molecule are significantly higher than the average ADP for protein atoms. This difference may suggest partial occupancy of dexamethasone, but is more likely to be a result of its positional variability between ESA molecules in the crystal. Refining dexamethasone at partial occupancy resulted in large positive difference-map peaks; therefore, we refined it at 100% occupancy. In addition to dexamethasone, one citrate molecule and one fatty-acid molecule were located in the structure. The citrate molecule is located inside the cleft between domains I and III, near DS9, at the same position as found in the ESA–testosterone complex; citrate was a major component of the crystallization cocktail. A fatty-acid molecule, which was likely to have been retained during the purification of ESA from blood, is located in fatty-acid site 8 (FA8). Very weak electron density is also observed in DS4, which does not allow any certainty in its interpretation. This density was accounted for by four UNX atoms. The electron density and the UNX atoms can be inspected interactively at https://molstack.bioreproducibility.org/project/view/gmvG1L8c66YgPpgqS0Zm/.

**Figure 2:**
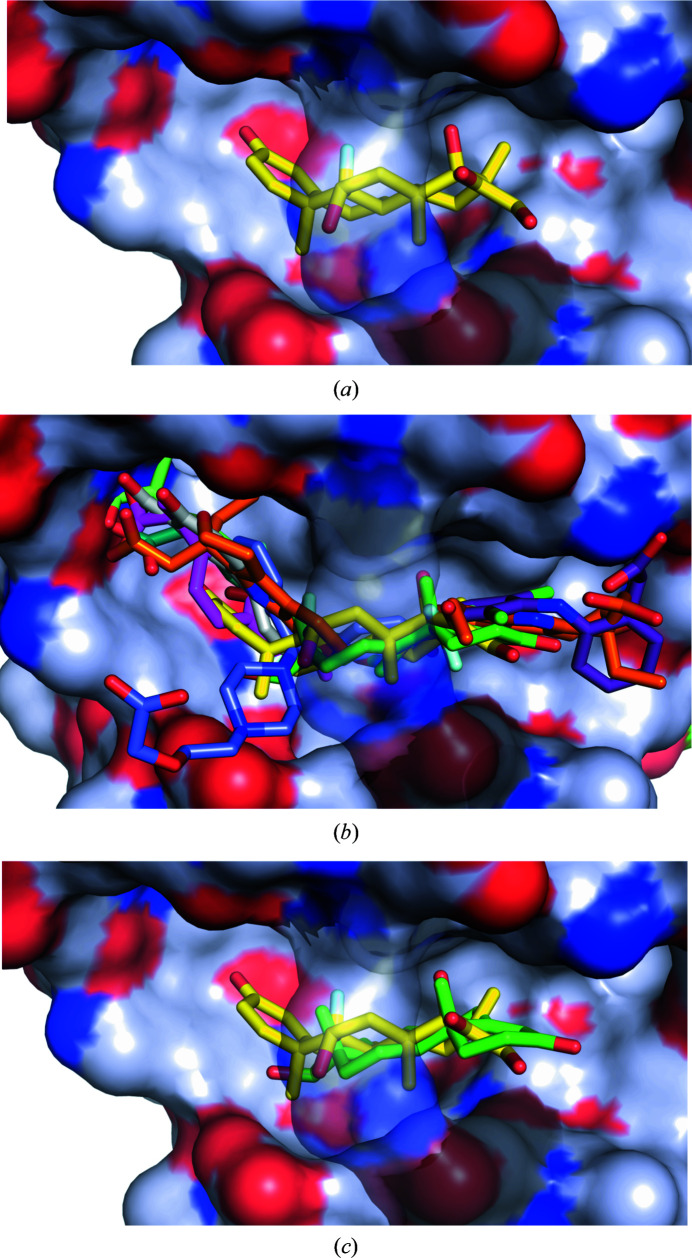
Hydrophobicity of DS7 and potential competition between dexamethasone and other drugs. (*a*) Dexamethasone (C atoms shown in yellow) bound to ESA. The color scheme for the protein surface is as follows: gray for the contribution from C atoms, red for that from O atoms and blue for that from N atoms. The link covering the cavity is formed by a salt bridge between Arg208 and Asp323 and is transparent in all panels for clarity. (*b*) Superposition of the complexes of serum albumin with all of the FDA-approved drugs known to bind to DS7: dexamethasone (yellow; PDB entry 6xk0), ibuprofen (pink; PDB entry 2bxg; Ghuman *et al.*, 2005 ▸), diflunisal (gray; PDB entry 2bxe; Ghuman *et al.*, 2005 ▸), cetirizine (blue; PDB entry 5dqf; Handing *et al.*, 2016 ▸), testosterone (green; PDB entry 6mdq; Czub *et al.*, 2019 ▸), halothane (gold; PDB entry 1e7b; Bhattacharya *et al.*, 2000 ▸), naproxen (pale green; PDB entry 4zbr; Sekula & Bujacz, 2016 ▸), 6-MNA (cyan; PDB entry 6u5a; Czub *et al.*, 2020 ▸), diclofenac (violet; PDB entry 6hn1; J. A. Talaj, A. Bujacz & G. Bujacz, unpublished work) and etodolac (orange; PDB entry 5v0v; Czub *et al.*, 2020 ▸). (*c*) Superposition of the ESA–dexamethasone (yellow; PDB entry 6xk0) and ESA–testosterone (green; PDB entry 6mdq; Czub *et al.*, 2019 ▸) structures. Dexamethasone and testosterone largely overlap at DS7. Both steroids are shown in stick representation with O atoms in red; dexamethasone is shown with C atoms in yellow and the F atom in cyan, while testosterone is shown with C atoms in green.

Fifteen residues are found within 5 Å of the dexamethasone molecule [Supplementary Fig. S1(*b*)]: Arg208, Ala209, Lys211, Ala212, Val215, Asp323, Leu326, Gly327, Leu330, Leu346, Arg347, Ala349, Lys350, Glu353 and Ala481 (which corresponds to Val482 in HSA). The side chains of Ala212, Val215, Leu326, Leu330, Leu346 and Ala349 and the hydrophobic part of Lys350 form a mostly hydrophobic surface at the inner side of the binding cavity, towards which the most hydrophobic part of the dexamethasone molecule is oriented [Fig. 2 ▸(*a*)]. The cavity is partially separated from the solvent by a strong salt bridge between Arg208 and Asp323, which bridges the IIA and IIB subdomains. In addition to the hydrophobic interactions, the drug molecule is stabilized by two hydrogen bonds between the O2 hydroxyl group and the NH2 atom of Arg208 and between the O3 hydroxyl group and the main-chain O atom of Arg208. In comparison, dexamethasone forms six hydrogen bonds to the glucocorticoid receptor (Raynor *et al.*, 2007 ▸). The significantly higher number of hydrogen bonds to the target of its action (the glucocorticoid receptor) than to the transport protein (albumin) is reflected in the respective binding constants: the dexamethasone dissociation constant for HSA has been reported as 58.8 µ*M* (Naik *et al.*, 2010 ▸) and 46.3 µ*M* (the smallest of the constants reported for four sites; Zhao *et al.*, 2009 ▸), both of which are four orders of magnitude higher than that for the glucocorticoid receptor: 4.6 n*M* (Ray *et al.*, 1999 ▸).

The high sequence identity/similarity between HSA and ESA (76.1%/86.2%) results in similar drug-binding properties of these albumins. Moreover, DS7 is classified as one of the most conserved drug-binding sites between ESA and HSA (Czub *et al.*, 2020 ▸). For example, the residue conservation in this site was shown to result in similar testosterone-binding properties of this site in albumin from both species (Czub *et al.*, 2019 ▸). Similarly, 14 of 15 residues involved in the binding of dexamethasone to ESA are conserved in HSA (Supplementary Fig. S1). Only one residue is different: Ala481 in ESA corresponds to a valine in HSA. This small hydrophobic-for-hydrophobic difference is unlikely to affect dexamethasone binding in HSA because it is a kind-for-kind change that does not introduce any clashes with the ligand. In fact, in the two structures of HSA that have a ligand bound to this site in the vicinity of this residue, complexes with diclofenac (PDB entry 4z69; Zhang *et al.*, 2015 ▸) and ibuprofen (PDB emtry 2bxg; Ghuman *et al.*, 2005 ▸), the valine residue turns away from the ligand. This conformation of valine makes its CB atom the closest atom to the ligand (3.4 Å), thus making valine very similar to alanine in terms of distances to the ligand. It should be noted that these two depositions have rather poor quality indicators according to the PDB validation report, and these models should be treated with caution. A complex of ovine serum albumin with diclofenac (PDB entry 6hn0; J. A. Talaj, A. Bujacz & G. Bujacz, unpublished), which has much better quality indicators, shows a different conformation of Val481 and a different position for diclofenac, which is located 6.6 Å from Val481 in this structure. However, even then the distance between Val481 and dexamethasone from a superposed ESA–diclofenac structure is sufficient (2.9 Å). Therefore, the conservation of amino-acid residues in DS7, which leads to essentially identical hydrophobic environments, allows us to hypothesize that dexamethasone binds to HSA in the same site as in ESA. This hypothesis is supported by the previously reported spectroscopic studies of the binding of dexamethasone by HSA and bovine serum albumin, which predicted that dexamethasone binds to the hydrophobic pocket around Trp214 inside the IIA subdomain (Naik *et al.*, 2010 ▸). In our ESA–dexamethasone structure, the distance between dexamethasone and the indole ring of Trp214 is about 12 Å.

Human serum albumin has multiple lysine and arginine residues that are known to be glycated (Anguizola *et al.*, 2013 ▸; Lee & Wu, 2015 ▸; Shaklai *et al.*, 1984 ▸; Nakajou *et al.*, 2003 ▸). Depending on the method, it is estimated that up to 6% of the HSA in a healthy human is glycated, while in diabetic patients these values are 2–5 times higher (Anguizola *et al.*, 2013 ▸; Shaklai *et al.*, 1984 ▸; Roohk & Zaidi, 2008 ▸). The residues in DS7 that are likely to undergo glycation are Arg208, Lys211 and Lys350 (Arg209, Lys212 and and Lys351, respectively, in HSA; Anguizola *et al.*, 2013 ▸). The glycation of Lys211 or Lys350 will not necessarily cause disruption of dexamethasone binding because these residues point outside the binding site towards the solution [Supplementary Fig. S1(*b*)]. However, the glycation of Arg208 is likely to prevent drug binding in this site owing to potential steric clashes and disruption of the binding site owing to possible elimination of the Arg208–Asp323 salt bridge, thus decreasing the binding capacity.

### 3.2. Competing compounds and albumin glycation may impair dexamethasone binding   

DS7 has been structurally characterized in human, equine, bovine, ovine, caprine and leporine albumin, and has been shown to bind testosterone, several nonsteroidal anti-inflammatory drugs (diclofenac, diflunisal, etodolac, ibuprofen, naproxen and 6-MNA), cetirizine and the general anesthetic halothane (Czub *et al.*, 2020 ▸). The site is formed by a large cavity, and some of the drugs shown to bind there occupy slightly different, yet overlapping, locations within the site [Fig. 2 ▸(*b*)]. Multiple drugs binding in the same binding site may result in drug–drug displacement: a drug may be displaced by another drug administered at a high concentration (Trainor, 2007 ▸; Bohnert & Gan, 2013 ▸). The increase in the free fraction of the displaced drug results in a decrease in its half-life and an increase in potential toxicity (Sułkowska *et al.*, 2004 ▸; McElnay & D’Arcy, 1983 ▸), which is especially concerning for drugs with small margins of safety (McElnay & D’Arcy, 1983 ▸). Although albumin is a major drug-transporting protein, structures of its complexes with only 32 FDA-approved drugs have been determined to date (Czub *et al.*, 2020 ▸), and it is unknown which of the drugs used in COVID-19 treatment would compete with dexamethasone. For example, colchicine (Colcrys), one of the drugs tested for effectiveness in COVID-19 patients, and theophylline (Elixophyllin), which is used in asthma and chronic obstructive pulmonary disease, have narrow therapeutic ranges and in the blood bind to albumin at 40% (US Food and Drug Administration, 2012 ▸; https://www.drugbank.ca/drugs/DB01394; https://www.drugbank.ca/drugs/DB00277), but their binding sites are unknown. Therefore, there is an urgent need to map the albumin binding sites for the most commonly prescribed drugs. Currently available structural data suggest potential competition between dexamethasone and drugs that bind to DS7 [Fig. 2 ▸(*b*)], some of which have already been reported to affect the way that dexamethasone works (https://www.drugs.com/monograph/dexamethasone.html; https://www.medicines.org.uk/emc/files/pil.5411.pdf).

Similarly, dexamethasone overlaps with the testosterone molecule [Fig. 2 ▸(*c*)], which is the only steroid for which a structure of a complex with albumin has been determined (Czub *et al.*, 2019 ▸). Testosterone is suspected of playing a critical role in driving the pronounced excess of COVID-19 lethality in male patients (Pozzilli & Lenzi, 2020 ▸), and low testosterone levels predict adverse clinical outcomes (Giagulli *et al.*, 2020 ▸). Critically ill male COVID-19 patients suffer from severe testosterone deficiency, which may or may not be caused by the disease. Competition of dexamethasone with testosterone for the same binding site may further exacerbate the effect of low testosterone by affecting its transport, especially if high doses of dexamethasone are administered.

Moreover, the location of the dexamethasone molecule suggests that dexamethasone transport may be affected by the glycation of albumin observed in diabetic patients. Diabetes is one of the major risk factors in COVID-19: 25.7% of males and 20% of females among admitted patients in one study had type II diabetes (Schroeder *et al.*, 2020 ▸). From the perspective of blood drug transport, diabetes presents an additional challenge owing to the glycation of drug-binding sites on albumin. Glycation, which is significantly increased in diabetic patients owing to elevated blood glucose levels, alters the drug-binding ability of albumin (Anguizola *et al.*, 2013 ▸). Four residues in DS7 can be glycated; among these, the glycation of Arg208 is likely to prevent drug binding in this site owing to potential steric clashes and disruption of the binding site by the elimination of the Arg208–Asp323 salt bridge, thus decreasing the binding capacity of albumin for dexamethasone.

### 3.3. Albumin and glucose levels of COVID-19 patients   

**Figure 3:**
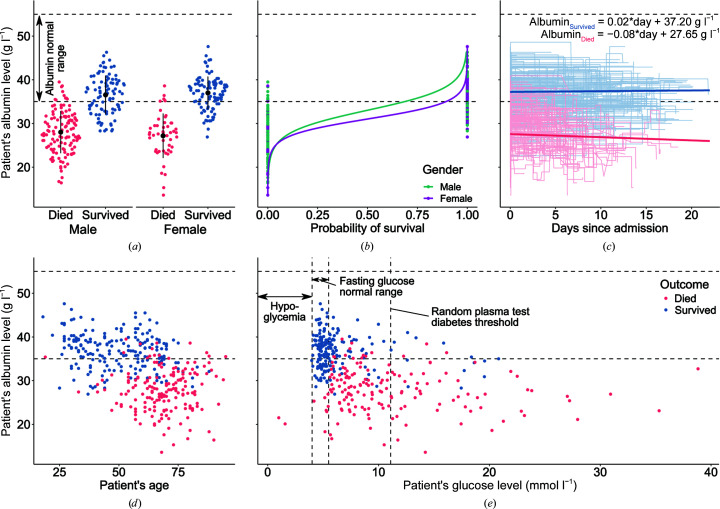
Albumin levels of 373 patients from Tongji Hospital, Wuhan, People’s Republic of China admitted between January 10 and February 18, 2020. (*a*) Violin strip charts of the final albumin levels of patients that died (red) and survived (blue), grouped by gender; means and standard deviations are overlaid. (*b*) Logistic regression showing the difference between the probability of survival for men (teal) and women (purple) at different albumin levels. (*c*) Line plots presenting the albumin levels of each patient over several blood samples taken during their time in the hospital; the median linear regression line between the first and last blood samples for each outcome group is overlaid. (*d*) Scatter plot presenting the relation between albumin level and age. (*e*) Scatter plot presenting the relation between final albumin and glucose levels. Source data are from Yan *et al.* (2020 ▸).

We have analyzed the albumin levels of COVID-19 patients admitted to Tongji Hospital, Wuhan, People’s Republic of China between January 10 and February 18, 2020 (Yan *et al.*, 2020 ▸). Out of 373 patients (222 males, 151 females), 174 died and 199 survived. The high mortality rate seen in this cohort (46.6%) stems from the fact that Tongji Hospital admitted a high rate of severe cases in Wuhan (Yan *et al.*, 2020 ▸). The albumin levels for both outcome groups were first positively tested for normality using Q–Q plots (Supplementary Fig. S1) and the Kolmogorov–Smirnov test (*W*_Died_ = 0.995, *W*_Survived_ = 0.991). The difference between mean albumin levels was found to be statistically significant according to Welch’s *t*-test (*p* < 0.001) and showed that patients that died were associated with much lower albumin levels (μ_Died_ = 27.8 g l^−1^) than those that survived (μ_Survived_ = 36.8 g l^−1^). The unadjusted odds ratio of surviving COVID-19 for albumin was 1.56 (95% CI 1.44–1.71, *p* < 0.001), whereas the odds ratio adjusted for gender, age and glucose level was 1.51 (95% CI 1.37–1.69, *p* < 0.001); detailed odds ratios for both models are presented in Supplementary Table S1. This means that each 1 g l^−1^ increase in the albumin level of the patient was associated with a 50% increased odds of surviving COVID-19. Other studies (Supplementary Table S3) have linked low levels of albumin to poor outcomes in COVID-19 (Chen *et al.*, 2020 ▸; Wu *et al.*, 2020 ▸; de la Rica *et al.*, 2020 ▸), but did not provide supporting blood-sample data that would allow full, time-series analyses of relations between different risk factors and patient survival rates. Fig. 3 ▸ shows patients’ albumin levels in relation to outcome, gender, time since admission, age and glucose level.

The majority of patients that died of COVID-19 had albumin levels that were not only lower than those of the patients that survived but were also below the normal range, which is usually defined as 35–55 g l^−1^ (Rifai, 2018 ▸). There is also a difference between the mortality rate among males (56.8%) and females (31.8%). Even though the albumin-level distributions for both genders have similar shapes, the proportions of survivors were not the same [Fig. 3 ▸(*a*)]. The adjusted odds ratio for surviving if a patient was female was 2.19 (95% CI 1.04–4.72, *p* < 0.05); however, the differences in survival chances corresponded mainly to high albumin levels [Fig. 3 ▸(*b*)]. This means that very low albumin levels were a strong predictor of death, regardless of gender. Moreover, the albumin levels of patients varied during their hospital stay [Fig. 3 ▸(*c*)]. The correlation between admission and outcome levels was statistically significant only for patients that died (*r*_Died_ = −0.22, *p* < 0.001;* r*_Survived_ = −0.07, *p* = 0.176), which indicates that patients that died from COVID-19 suffered a decrease in albumin levels during their hospital stay. Furthermore, age was also a risk factor [Fig. 3 ▸(*d*)], with each year decreasing the odds of survival by 8% (odds ratio 0.92, 95% CI 0.89–0.94, *p* < 0.001), which is in agreement with previous studies showing age to be a predictor of mortality in COVID-19 (Zhou *et al.*, 2020 ▸). Finally, it can be noticed that high glucose levels were also a significant risk factor [Fig. 3 ▸(*e*)]. The adjusted odds ratio of surviving COVID-19 for glucose was 0.89 (95% CI 0.81–0.96, *p* < 0.001), which means that every 1 mmol l^−1^ increase in glucose level was associated with a 12% lower odds of surviving. This finding is in agreement with previous studies that have shown that diabetes, which is associated with high glucose levels, is another COVID-19 risk factor (Schroeder *et al.*, 2020 ▸; Zhou *et al.*, 2020 ▸). Moreover, isolated hyperglycemia is common following trauma, illness or infection, and manifests as transient states of insulin resistance which can resolve post-illness (Chen *et al.*, 2012 ▸; Eakins, 2009 ▸).

Hypoalbuminemia in COVID-19 patients has previously been described and was found to be a significant risk factor for increased mortality (Huang, Cheng *et al.*, 2020 ▸; Ramadori, 2020 ▸; Aziz *et al.*, 2020 ▸). However, the role of serum albumin and its blood level in COVID-19 are not yet completely understood. Association of low albumin with poor outcomes is not unique to COVID-19: low levels are associated with inflammation (Soeters *et al.*, 2019 ▸) and cancer (Nazha *et al.*, 2015 ▸), and are generally regarded as detrimental in many medical conditions (Gatta *et al.*, 2012 ▸; Ulldemolins *et al.*, 2011 ▸). Hypoalbuminemia at triage has been found to predict increased 30-day mortality among all acutely admitted hospital patients (Jellinge *et al.*, 2014 ▸) and to be a risk factor for acute respiratory distress syndrome (ARDS; Wu *et al.*, 2020 ▸). One potential cause for low albumin levels in these COVID-19 cases is prior comorbidities, such as malnutrition or liver disease, which predispose an affected patient to a more severe course of the illness. A second potential cause is that albumin levels decrease with age [Fig. 3 ▸(*d*)] (Weaving *et al.*, 2016 ▸). Lastly, the disease itself can decrease albumin levels via a reduction in albumin synthesis owing to reduced food intake (Ramadori, 2020 ▸), the virus attacking the bile ducts and possibly other hepatic cells (Xu *et al.*, 2020 ▸), or the hepatotoxicity of a COVID-19-induced cytokine storm (Huang, Li *et al.*, 2020 ▸; Ramadori, 2020 ▸). Our analysis shows only a minor albumin decline in patients that died [Fig. 3 ▸(*c*)], suggesting that this cause is unlikely to be a major one. However, it cannot be excluded that a significant decrease in albumin levels happened in the early stages of COVID-19, prior to the admission of the patients to the hospital. This may have happened at Tongji Hospital, which admitted mostly severely ill patients. In any case, albumin levels may serve as a reliable predictor of COVID-19 severity. Therefore, serum albumin infusion aimed at correcting hypoalbuminemia, as used in severe liver damage, should be considered for COVID-19 patients, as has also been proposed elsewhere (Garcia-Martinez *et al.*, 2013 ▸; Mani Mishra *et al.*, 2020 ▸).

### 3.4. Dexamethasone effectiveness in COVID-19 may be affected by hypoalbuminemia, drug–drug displacement and diabetes   

Our analysis of structural and clinical data demonstrates that serum albumin plasma level, competing drugs and albumin glycation are important clinical variables that may influence the effectiveness of dexamethasone in treating COVID-19 patients. Each of these three factors decreases the binding capacity of albumin, which can have two adverse implications on dexamethasone blood transport: a shorter half-life of the drug and potential toxicity at its peak concentration. The average half-life of dexamethasone in pneumonia patients was reported to be 6.9 h for 6 mg oral administration and 9.0 h for 4 mg intravenous injection (Spoorenberg *et al.*, 2014 ▸). Because albumin acts as storage for drugs, its reduced binding capacity is likely to result in a shorter half-life of dexamethasone, decreasing its therapeutic action. Moreover, reduced binding capacity increases the risk of dexamethasone toxicity because the maximum concentration of unbound dexamethasone increases. Side effects of dexamethasone are common, and low albumin levels have been observed to significantly increase the risk of its toxicity (Weissman *et al.*, 1987 ▸; Kostaras *et al.*, 2014 ▸). Therefore, future clinical trials should consider a modified dexamethasone regimen in COVID-19 treatment when (i) patients suffer from hypoalbuminemia, (ii) co-administered drugs compete with dexamethasone for DS7 or (iii) DS7 is compromised by glycation owing to diabetes. Notably, the presence of all three of these conditions may have a strong compounding effect. Dexamethasone regimen modifications could involve adjusting for the patient’s weight and/or splitting the daily dose into multiple doses to achieve a sufficient plasma level of dexamethasone over time while reducing the possible peak concentration, especially if doses of higher than 6 mg are used (Pan *et al.*, 2016 ▸). The idea that hypoalbuminemia may have an effect on optimal drug dosing is not new: for example, this effect has been studied for antibiotic dosing and was found to be an important consideration (Ulldemolins *et al.*, 2011 ▸). The need to adapt dosing regimens rationally based on the pharmacokinetic/pharmacodynamic characteristics of various drugs has also been reported (Roberts *et al.*, 2013 ▸). Another feasible way of bypassing the limitations of vascular transport of dexamethasone would be to administer it via inhalers (Halpin, Singh *et al.*, 2020 ▸); it has been suggested that such steroid use may play a protective role in reducing vulnerability to COVID-19 (Halpin, Faner *et al.*, 2020 ▸). Once the albumin levels of patients in the RECOVERY trial are released, a further analysis should be performed to establish whether there is a correlation between the effectiveness of dexamethasone intervention and hypoalbuminemia, drug co-administration and serum albumin glycation.

## 4. Conclusions   

The presented molecular analysis of the binding of dexamethasone to serum albumin in combination with the risk factors identified from the clinical data analyzed here and elsewhere offer promising strategies for maximizing the effectiveness of dexamethasone in the treatment of severe COVID-19. We propose that further research should be performed to evaluate adjusting the dexamethasone dosing regimen as a strategy for patients with severe COVID-19 and with hypoalbuminemia, diabetes or co-administration of large doses of drugs that bind to DS7. Moreover, oral inhalers should be assessed as an alternative route of dexamethasone administration, which avoids the limitations of vascular transport. Our analysis also strengthens the case for the evaluation of albumin infusion aimed at the correction of hypoalbuminemia. Guidelines for the treatment of COVID-19 are still rapidly emerging (Lane & Fauci, 2020 ▸) and will remain critical even after a vaccine is developed, and dexamethasone will be likely to play a part in that treatment.

## Supplementary Material

PDB reference: serum albumin, complex with dexamethasone, 6xk0

Diffraction images.: https://doi.org/10.18430/m3.irrmc.5571

Supplementary Figures and Tables. DOI: 10.1107/S2052252520012944/be5285sup1.pdf

## Figures and Tables

**Table 1:** Data-collection, structure-refinement and structure-quality statistics Values in parentheses are for the highest resolution shell. The Ramachandran plot statistics were calculated by *MolProbity*.

PDB code	6xk0
Diffraction data DOI	https://doi.org/10.18430/m3.irrmc.5571
Data-collection statistics	
Data-collection date	6 July 2011
Resolution (Å)	50.00–2.40 (2.44–2.40)
Beamline	21-ID-F
Wavelength (Å)	0.979
Space group	*P*6_1_
Unit-cell parameters (Å)	*a* = *b* = 95.0, *c* = 143.6
Protein chains in the asymmetric unit	1
Completeness (%)	99.8 (98.3)
No. of unique reflections	28870 (1412)
Multiplicity	4.9 (3.5)
〈*I*〉/〈σ(*I*)〉	15.3 (1.3)
CC_1/2 _	(0.61)
*R*_merge_	0.110 (1.053)
*R*_meas_	0.123 (1.221)
Refinement statistics	
*R*_work_/*R*_free_	0.203/0.249
R.m.s.d., bond lengths (Å)	0.002
R.m.s.d., bond angles (°)	1.1
Mean ADP (Å^2^)	57
Mean ADP for dexamethasone (Å^2^)	90.5
No. of protein atoms	4589
RSR for dexamethasone	0.161
RSCC for dexamethasone	0.894
Mean ADP for protein (Å^2^)	58
No. of water molecules	228
Mean ADP for water molecules (Å^2^)	39
Clashscore	2.71
*MolProbity* score	1.25
Rotamer outliers (%)	0.60
Ramachandran outliers (%)	0.00
Ramachandran favored (%)	96.88
