# Supplementary material for: Molecular determinants of vascular transport of dexamethasone in COVID-19 therapy
Source: IUCrJ. 2020 Sep 29;7(Pt 6):1048–58. doi: 10.1107/S2052252520012944 (PMC7553145; doi:10.1107/S2052252520012944)
Supplement: Supplementary file 1 [file m-07-01048-sup1.pdf]

IUCrJ

Volume 7 (2020)

Supporting information for article:

Molecular determinants of vascular transport of dexamethasone in COVID-19 therapy

Ivan G. Shabalin, Mateusz P. Czub, Karolina A. Majorek, Dariusz Brzezinski, Marek Grabowski, David R. Cooper, Mateusz Panasiuk, Maksymilian Chruszcz and Wladek Minor

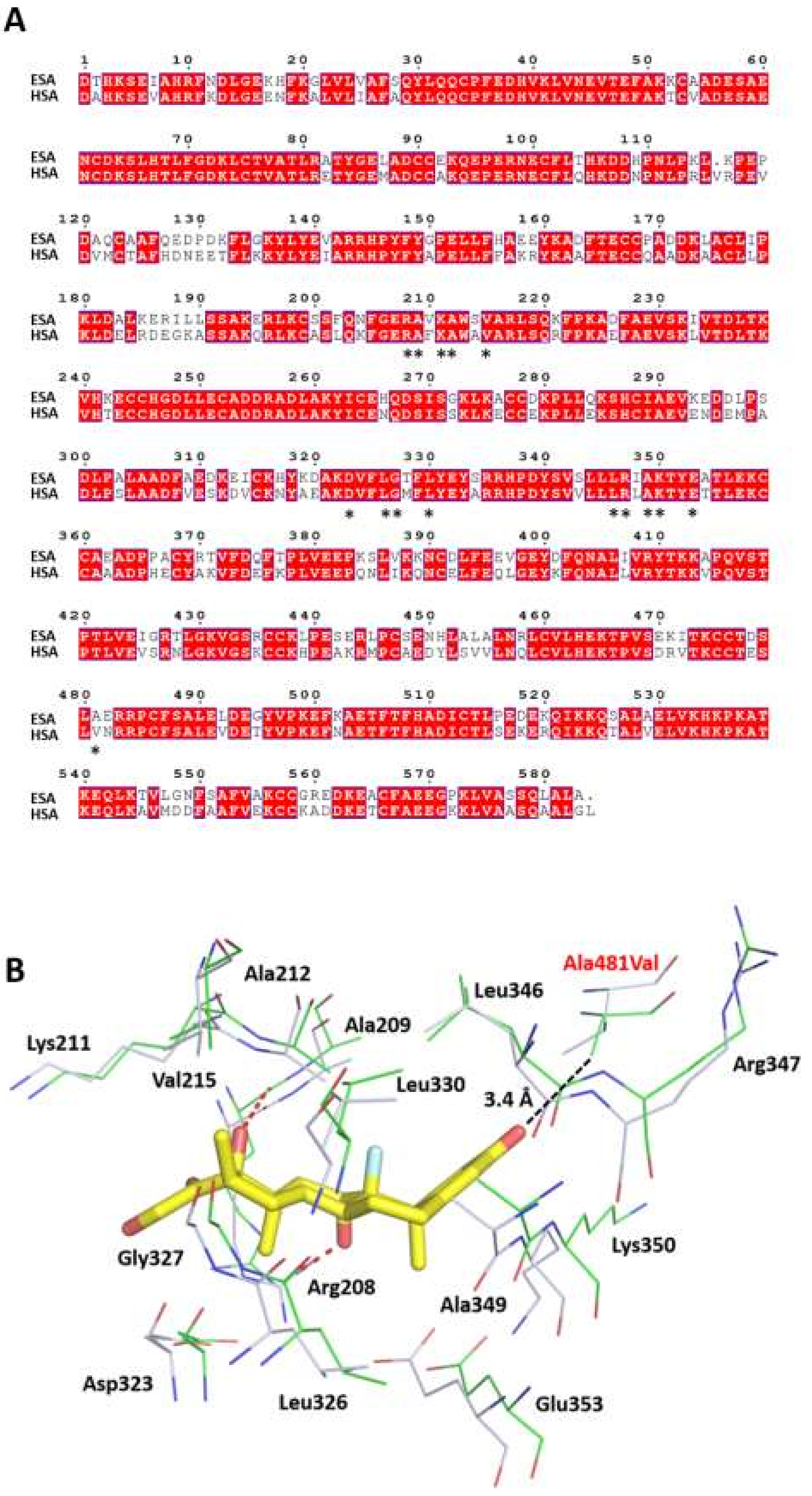
A
ESA
HSA
B
Ala212
Ala481Val
Leu346
Ala209
Lys211
Arg347
Val215
Leu330
3.4 Å
Lys350
Gly327
Arg208
Ala349
Asp323
Leu326
Glu353

Figure S1 Comparison of ESA and HSA. (**A**) Alignment of ESA and HSA sequences. Identical residues are highlighted in red; residues involved in dexamethasone binding to DS7 in ESA and analogous residues in HSA are marked with stars. (**B**) Superposition of DS7 binding dexamethasone in ESA (PDB ID: 6XK0) and analogous site in HSA (PDB ID: 4K2C). Dexamethasone is shown in stick representation with carbon atoms in yellow, oxygen atoms in red and fluoride atom in cyan. Residues within 5 Å are shown in line representation, residues from ESA are shown with carbon atoms in green while residues from HSA are shown with carbon atoms in gray, oxygen and nitrogen atoms are red and blue, respectively. Residue numbers correspond to positions in ESA. Ala481 is the only residue at this site that differ in HSA (Val in HSA). Hydrogen bonds are shown as red dashes.

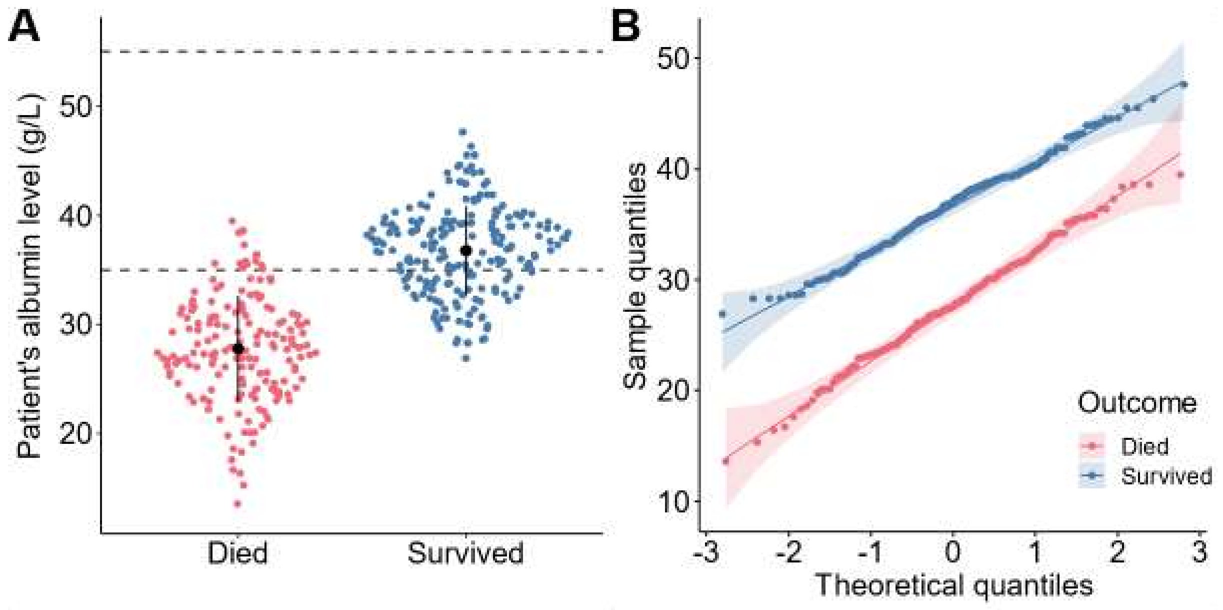

Figure S2 Distribution of COVID-19 patients grouped by outcome. (A) Violin strip charts (sina plots) of patients that died (red) and (survived), after being admitted to Tongji Hospital. (B) Q-Q plot for the albumin levels of patients that died and survived COVID-19. The shaded areas represent 95% confidence intervals.

Table S1 Associations between gender, age, glucose level, albumin level and survival or death in univariate and multivariate logistic regression analyses.

Analysis	Odds ratio of survival (95% CI)	p-values
Univariate analysis (unadjusted models)		
Albumin level	1.56 (1.44–1.71)	$p < 0.001$
Female	2.82 (1.83–4.37)	$p < 0.001$
Age	0.90 (0.88–0.92)	$p < 0.001$
Glucose level	0.76 (0.70–0.81)	$p < 0.001$
Multivariate analysis (adjusted model)		
Albumin level	1.51 (1.37–1.69)	$p < 0.001$
Female	2.19 (1.04–4.72)	$p < 0.05$
Age	0.92 (0.89–0.94)	$p < 0.001$
Glucose level	0.89 (0.81–0.96)	$p < 0.01$

Table S2 RMSD values [Å] for aligned Cα atoms of ESA-steroid complexes and ligand-free ESA and HSA structures.

	ESA-dexamethasone (PDB ID: 6XK0)	ESA-testosterone (PDB ID: 6MDQ)	ESA-ligand free (PDB ID: 3V08)	HSA-ligand free (PDB ID: 4K2C)
ESA-dexamethasone (PDB ID: 6XK0)	-	1.1	0.4	1.6
ESA-testosterone (PDB ID: 6MDQ)	1.1	-	1.1	1.6
ESA-ligand free (PDB ID: 3V08)	0.4	1.1	-	1.7
HSA-ligand free (PDB ID: 4K2C)	1.6	1.6	1.7	-

Table S3 Comparison of mean (s.d) of albumin levels and patient outcomes in different studies. N denotes the sample size of the study. ICU: Intensive Care Unit, ARDS: Acute Respiratory Distress Syndrome.

Country	N	Group 1 (g/L)	Group 2 (g/L)	Study
Spain	48	ICU: 29.0 (5.2)	Non-ICU: 39.2 (4.2)	(Rica *et al.*, 2020)
China*	21	Severe: 29.6 (28.6–33.0)	Moderate: 37.2 (35.8–38.8)	(Chen *et al.*, 2020)
China*	201	ARDS: 30.4 (27.15–33.35)	Non-ARDS: 33.7 (30.95–36.30)	(Wu *et al.*, 2020)
China*†	2623	Died: 31.1 (27.9–34.2)	Non-critical: 36.6 (33.2–40.4)<br>Critical: 32.2 (29.6–35.7)	(Huang *et al.*, 2020)
China‡	910	Severe: 35.0 (2.4)	Non-severe: 40.5 (2.2)	(Aziz *et al.*, 2020)
China	373	Died: 27.8 (4.9)	Survived: 36.8 (4.1)	current

*Albumin level characterized by the median and quartiles (Q1–Q3); †Albumin levels measured at admission; ‡Meta-analysis of data form different hospitals

References

Anguizola, J., Matsuda, R., Barnaby, O. S., Hoy, K. S., Wa, C., DeBolt, E., Koke, M. & Hage, D. S. (2013). *Clin. Chim. Acta*. **425**, 64–76.

Aziz, M., Fatima, R., Lee-Smith, W. & Assaly, R. (2020). *Crit. Care*. **24**, 255.

Chen, G., Wu, D., Guo, W., Cao, Y., Huang, D., Wang, H., Wang, T., Zhang, X., Chen, H., Yu, H., Zhang, X., Zhang, M., Wu, S., Song, J., Chen, T., Han, M., Li, S., Luo, X., Zhao, J. & Ning, Q. (2020). *J. Clin. Invest.* **130**, 2620–2629.

Holm, L. (2019). *Bioinformatics*. **35**, 5326–5327.

Huang, W., Li, C., Wang, Z., Wang, H., Zhou, N., Jiang, J., Ni, L., Zhang, X. A. & Wang, D.-W. (2020). *Sci. China Life Sci.*

Lee, P. & Wu, X. (2015). *Curr. Pharm. Des.* **21**, 1862–1865.

Nakajou, K., Watanabe, H., Kragh-Hansen, U., Maruyama, T. & Otagiri, M. (2003). *Biochim. Biophys. Acta*. **1623**, 88–97.

Rica, R. de la, Borges, M., Aranda, M., Castillo, A. del, Socias, A., Payeras, A., Rialp, G., Socias, L., Masmiquel, L. & Gonzalez-Freire, M. (2020). *MedRxiv*. 2020.05.07.20094987.

Roohk, H. V. & Zaidi, A. R. (2008). *J. Diabetes Sci. Technol.* **2**, 1114–1121.

Shaklai, N., Garlick, R. L. & Bunn, H. F. (1984). *J. Biol. Chem.* **259**, 3812–3817.

Wu, C., Chen, X., Cai, Y., Xia, J., Zhou, X., Xu, S., Huang, H., Zhang, L., Zhou, X., Du, C., Zhang, Y., Song, J., Wang, S., Chao, Y., Yang, Z., Xu, J., Zhou, X., Chen, D., Xiong, W., Xu, L., Zhou, F., Jiang, J., Bai, C., Zheng, J. & Song, Y. (2020). *JAMA Intern. Med.*
